# Predictive modeling of perioperative blood transfusion in lumbar posterior interbody fusion using machine learning

**DOI:** 10.3389/fphys.2023.1306453

**Published:** 2023-12-22

**Authors:** Fang-Fang Lang, Li-Ying Liu, Shao-Wei Wang

**Affiliations:** ^1^ School of Public Health, Shanxi Medical University, Taiyuan, China; ^2^ Department of Orthopedics, The Second Hospital of Shanxi Medical University, Taiyuan, China

**Keywords:** lumbar posterior interbody fusion, perioperative blood transfusion, machine learning, interpretability, risk factors

## Abstract

**Background:** Accurate estimation of perioperative blood transfusion risk in lumbar posterior interbody fusion is essential to reduce the number, cost, and complications associated with blood transfusions. Machine learning algorithms have the potential to outperform traditional prediction methods in predicting perioperative blood transfusion. This study aimed to construct a machine learning-based perioperative transfusion risk prediction model for lumbar posterior interbody fusion in order to improve the efficacy of surgical decision-making.

**Methods:** We retrospectively collected clinical data on 1905 patients who underwent lumbar posterior interbody fusion surgery at the Second Hospital of Shanxi Medical University between January 2021 and March 2023. All the data was randomly divided into a training set and a validation set, and the “feature_importances” method provided by eXtreme Gradient Boosting (XGBoost) algorithm was applied to select statistically significant features on the training set to establish five machine learning prediction models. The optimal model was identified by utilizing the area under the curve (AUC) and the probability calibration curve on the validation set. Shapley additive explanations (SHAP) and local interpretable model-agnostic explanations (LIME) were employed for interpretable analysis of the optimal model.

**Results:** In the postoperative outcomes of patients, the number of hospital days in the transfusion group was longer than that in the non-transfusion group. Additionally, the transfusion group experienced higher total hospital costs, 90-day readmission rates, and complication rates within 90 days after surgery than the non-transfusion group. A total of 9 features were selected for the models. The XGBoost model performed best with an AUC value of 0.958. The SHAP values showed that intraoperative blood loss, intraoperative fluid infusion, and number of fused segments were the top 3 most important features affecting perioperative blood transfusion in lumbar posterior interbody fusion. The LIME algorithm was used to interpret the individualized prediction.

**Conclusion:** Surgery, ASA class, levels fused, total intraoperative blood loss, operative time, and preoperative Hb are viable predictors of perioperative blood transfusion in lumbar posterior interbody fusion. The XGBoost model has demonstrated superior predictive efficacy compared to the traditional logistic regression model, making it a more effective decision-making tool for perioperative blood transfusion.

## Introduction

Lumbar interbody fusion is a classic surgical procedure widely utilized both domestically and internationally for the treatment of degenerative lumbar spine disorders such as lumbar disc herniation, lumbar spinal stenosis, and lumbar spondylolisthesis (Ristagno et al., 2018; Tang et al., 2020). This type of surgery provides spinal stability by eliminating nerve compression, which can significantly relieve nerve root symptoms and pain (Guan et al., 2022; Xiong et al., 2023). However, blood transfusions are frequently required during the perioperative period since the surgical procedure is highly intrusive and necessitates complete dissection of the paravertebral soft tissue as well as excision of the lamina and intervertebral disc (Suk et al., 1997; Nuttall et al., 2000; Salehi et al., 2004; Willner et al., 2016). Previous studies have shown that perioperative blood transfusion increases the risk of postoperative complications, including infection, fever, transmission of blood-borne diseases, pneumonia, and incisional complications, which is a huge economic burden for the healthcare system (Shander et al., 2010; Aoude et al., 2016a; Kato et al., 2016). Therefore, clarifying the predictors of perioperative blood transfusion can better identify high-risk patients for early intervention to reduce the number of transfusions and related complications.

Artificial intelligence (AI) has recently been increasingly applied in various fields of medicine (Chaofan et al., 2022; Zhen et al., 2022; FukHay et al., 2023). Machine learning (ML), a subfield of AI, can automatically predict the output through algorithms based on the characteristics of input data. Compared with traditional statistical methods, ML can process big data more accurately, thereby significantly improving the accuracy of diagnosis and prognosis prediction ability (Khan et al., 2020; Bellini et al., 2022; Ren et al., 2022). Although logistic regression (LR) have been extensively utilized in clinical disease prediction research and are comprehensible to clinical workers, it also has some limitations including poor classification accuracy, underfitting, and prediction efficacy that is easily affected by missing data (Boehm et al., 2016; Mistry et al., 2017). Most studies on predicting perioperative blood transfusion in lumbar interbody fusion currently use traditional logistic regression methods to analyze risk factors (Wang et al., 2021a; Liu et al., 2021; Chen et al., 2022). However, consensus on the risk factors and prediction accuracy remains insufficient. The objective of this study was to develop interpretable machine learning prediction models to improve the efficacy of perioperative blood transfusion risk prediction for lumbar posterior interbody fusion and to provide clinicians with better surgical transfusion decision making.

## Materials and methods

### Study population

Electronic medical records for patients who underwent lumbar interbody fusion surgery at the Second Hospital of Shanxi Medical University were collected retrospectively. Inclusion criteria: 1) patients who met the diagnosis of lumbar degenerative disease and failed to respond to standard conservative treatment; 2) posterior lumbar interbody fusion (PLIF) or transforaminal lumbar interbody fusion (TLIF); 3) age ≥40 years. Exclusion criteria: 1) minimally invasive surgery; 2) lumbar spine tumour; 3) lumbar spine tuberculosis; 4) brucellosis; 5) lumbar scoliosis deformity; 6) lumbar fracture and dislocation; 7) cervical or thoracic surgery; 8) autologous blood transfusion; 9) preoperative blood transfusion (Wang et al., 2021a). The study waived informed consent and was approved by the Ethics Committee of the Second Hospital of Shanxi Medical University (Ethical approval code: 2023YX235). Between January 2021 and March 2023, there were 1,987 patients satisfied the inclusion and exclusion criteria, with 82 patients who had a missing clinical data rate exceeding 20% being excluded. Ultimately, 1,905 patients were enrolled in this trial, consisting of 953 (50%) males and 952 (50%) females, all of whom underwent standard lumbar interbody fusion. Blood transfusion was defined as intraoperative or postoperative transfusion of at least 1 U of allogeneic suspension-concentrated red blood cells.

### Data collection and processing

Based on a literature search and experience in clinical practice (Lou et al., 2022), this study documented patients’ clinical information in electronic medical records, including gender, age, body mass index (BMI), duration of disease, concomitant diseases, Previous history, preoperative laboratory tests, surgery, American Stroke Association (ASA) class, levels fused, intraoperative fluid infusion volume, total intraoperative estimated blood loss, intraoperative urine volume, operative time, time to surgery, tranexamic acid use, preoperative functional status, and postoperative outcomes data, which included length of stay, total hospitalization cost, 90-day readmission, and postoperative complications (within 90 days). According to the data collected, subjects with more than 20% missing information were eliminated, and subjects with less than 20% missing information were filled. Continuous variables were filled using the conditional mean filling method, whereas categorical variables were filled using the random interpolation filling method.

### Statistical analysis

SPSS 26.0 statistical software was used to analyze the differences in characteristics between the blood transfusion group and the non-blood transfusion group. Continuous characteristics were expressed as mean ± SD and compared with the use of Student’s t-test or the Mann Whitney U test. Categorical characteristics were expressed as numbers and percentages and compared using Pearson’s chi-squared test or Fisher’s exact test. Variables with a *p*-value less than 0.05 in univariate analysis were entered into a multivariate logistic regression analysis to ascertain the independent risk factors for perioperative blood transfusion in posterior lumbar interbody fusion. Statistical significance was considered for *p* < 0.05.

### Development of predictive models

The research cohort was randomly divided into a training set (80%) and a validation set (20%). In the training set, features with *p* values less than 0.05 in the univariate analysis were chosen as alternative features, and the “feature_importances” method provided by the XGBoost algorithm was applied for feature selection (Chen and Guestrin, 2016). XGBoost is based on the gain of the structure scores to determine which feature to choose as the segmentation point, and the importance of this feature depends on the sum of its number of occurrences in all tree structures. When an attribute is widely used in the model to build a decision tree, its importance increases accordingly. One advantage of using the XGBoost algorithm for feature selection is that the importance score of each feature can be calculated relatively intuitively after building the promotion tree. The predictors with the highest feature importance scores are entered into 5 ML algorithms, namely, Extreme Gradient Boosting (XGB), random forest (RF), support vector machine (SVM), naïve bayes (NB) and artificial neural network (ANN), to build the prediction model. Ten-fold cross-validation and grid search techniques were utilized to fine-tune the optimal parameters of the model, and the conclusive updated parameters following several iterations were deemed as the ideal configuration for the current model. The algorithm’s parameters are provided in (Supplementary Table S1). Model performance was assessed on a validation set using AUC, accuracy, recall, specificity, F1-Scores and probability calibration curves (Moons et al., 2019). SHAP (Lundberg et al., 2020; Wang et al., 2021b) and LIME (Molnar, 2020) were used to explainably analyze the optimal model. All data analysis and construction were conducted using Python 3.10.9. LR, XGBoost, RF, SVM and NB were established and trained using the scikit-learn (1.2.2) package within Python. The ANN model was mainly constructed using the keras (2.12.0) and tensorflow (2.12.0) frameworks. SHAP explanatory analysis was conducted using shap (0.42.1), and LIME analysis utilized lime (0.42.1) and jupyter (1.0.0).

## Results

### Demographic baseline characteristics

A total of 1905 valid samples were included and a few samples had missing values. Missing values for continuous variables were imputed using the conditional mean method, while missing values for categorical variables were imputed using the random interpolation method. In the transfusion group, there were 360 patients (18.9%) with an average age of (65.0 ± 9.9) years, while the non-transfusion group comprised 1545 patients (81.1%) with an average age of (60.7 ± 9.5) years. Table 1 shows the comparison of preoperative and intraoperative characteristics data of patients between the two groups by univariate analysis. For postoperative outcomes, the transfusion group had a longer length of stay compared to the non-transfusion group. Additionally, the total hospitalization cost, 90-day readmission rate, and incidence of complications within 90 days after operation were higher in the transfusion group. All differences had *p*-values below 0.05, as shown in Table 2. The results of multivariate logistic regression analyses showed a higher risk of perioperative blood transfusion in lumbar posterior interbody fusion patients with hypertension, PLIF Surgery, ASA class ≥ III, levels fused ≥2, higher total intraoperative blood loss, longer operative time, and lower preoperative hemoglobin (Hb)、preoperative sodium、preoperative albumin, as shown in Table 3.

**TABLE 1:** Comparison of preoperative and intraoperative data of the two groups of patients.

Variable	Non-transfusion (N = 1545)	Transfusion (N = 360)	t/χ^2^	*P*
Sex			0.230	0.632
Female	777 (50.3%)	176 (48.9%)		
Male	768 (49.7%)	184 (51.1%)		
Age (years)	60.7 ± 9.5	64.9 ± 9.9	7.438	<0.001
BMI (kg/m^2^)	25.1 ± 3.5	25.7 ± 3.8	2.324	0.021
Duration of disease (months)	53.5 ± 71.9	65.2 ± 79.5	2.554	0.011
Comorbidities (%)				
Hypertension	520 (33.7%)	182 (50.6%)	35.828	<0.001
Diabetes mellitus	184 (11.9%)	70 (19.4%)	14.346	0.001
Coronary heart disease	79 (5.1%)	42 (11.7%)	21.080	0.024
Previous history (%)				
Surgical history	604 (39.1%)	174 (48.3%)	10.316	0.001
Blood transfusion	31 (2.0%)	12 (3.3%)	2.330	0.127
Allergies	178 (11.5%)	47 (13.1%)	0.660	0.417
Smoking	420 (27.2%)	91 (25.3%)	0.541	0.462
Alcohol	281 (18.2%)	44 (12.2%)	7.343	0.007
Preoperative laboratory tests				
WBC	6.4 ± 1.8	6.4 ± 2.1	−0.193	0.847
RBC	4.6 ± 0.5	4.5 ± 0.5	−5.154	<0.001
Hb	142.6 ± 15.4	137.2 ± 19.0	−4.998	<0.001
HCT	0.4 ± 0.0	0.4 ± 0.1	−4.834	<0.001
PLT	229.8 ± 60.7	229.2 ± 82.1	−0.142	0.887
PT	13.7 ± 0.9	13.9 ± 1.2	4.168	<0.001
INR	1.0 ± 0.1	1.0 ± 0.1	3.784	<0.001
FIB	2.8 ± 0.6	2.9 ± 0.7	1.687	0.09
APTT	30.7 ± 3.1	30.8 ± 3.2	0.783	0.434
Sodium	140.6 ± 2.2	140.3 ± 3.0	−2.114	0.035
Calcium	2.3 ± 0.2	2.3 ± 0.3	−0.379	0.705
Albumin	40.5 ± 3.3	39.7 ± 3.8	−4.042	<0.001
Surgery			76.130	<0.001
TLIF	489 (31.7%)	32 (8.9%)		
PLIF	1056 (68.3%)	328 (91.1%)		
ASA class			75.829	<0.001
Ⅰ、Ⅱ	1291 (83.6%)	227 (63.1%)		
≥Ⅲ	254 (16.4%)	133 (36.9%)		
Levels fused			326.961	<0.001
1	1207 (78.1%)	116 (32.2%)		
2	307 (19.9%)	186 (51.7%)		
≥3	31 (2.0%)	58 (16.1%)		
Intraoperative FIV (mL)	1851.0 ± 419.1	2137.6 ± 547.5	9.316	<0.001
Total intraoperative EBL (mL)	235.2 ± 149.4	614.9 ± 309.7	22.655	<0.001
Intraoperative UV (mL)	392.2 ± 190.8	494.4 ± 259.3	7.050	<0.001
Operative time (min)	134.2 ± 33.3	169.8 ± 42.6	14.854	<0.001
Time to surgery (days)	4.3 ± 2.3	4.8 ± 2.8	3.270	0.001
Tranexamic acid use			0.859	0.354
Yes	252 (16.3%)	66 (18.3%)		
No	1293 (83.7%)	294 (81.7%)		
Preoperative functional status			0.48	0.488
Independent	237 (15.3%)	50 (13.9%)		
Dependent	1308 (84.7%)	310 (86.1%)		

WBC, white blood cell count; RBC, red blood cell count; Hb, Hemoglobin; HCT, hematocrit; PLT, platelet; PT, prothrombin time; INR, international normalized ratio; FIB, fbrinogen; APTT, activated partial thromboplastin time; FIV, fluid infusion volume; EBL, estimated blood loss; UV, urine volume.

**TABLE 2:** Comparison of postoperative outcomes of the two groups of patients.

	Non-transfusion (N = 1545)	Transfusion (N = 360)	t/χ^2^	*P*
Length of stay (days)	9.0 ± 3.2	10.2 ± 3.8	5.828	<0.001
Total hospitalization cost (yuan)	68572.6 ± 14721.4	78928.4 ± 21864.8	8.546	<0.001
90-day readmission	23 (1.5%)	11 (3.1%)	4.089	0.043
Postoperative complications (within 90 days)	31 (2.0%)	18 (5.0%)	10.440	0.001

**TABLE 3:** Multivariate logistic regression analyses for risk factors of blood transfusion.

Risk factors	β	SE	Wald value	*P*	OR (95%CI)
Hypertension	0.479	0.199	5.832	0.016	1.615 (1.095–2.384)
Surgery	0.995	0.287	12.000	0.001	2.704 (1.540–4.748)
ASA class	0.936	0.220	18.176	<0.001	2.551 (1.658–3.923)
Levels fused					
2	1.063	0.217	24.085	<0.001	2.896 (1.894–4.429)
≥3	1.657	0.370	20.064	<0.001	5.242 (2.539–10.822)
Total intraoperative EBL	0.008	0.001	173.783	<0.001	1.008 (1.007–1.009)
Operative time	0.010	0.003	10.110	0.001	1.010 (1.004–1.016)
Preoperative Hb	−0.036	0.006	31.876	<0.001	0.965 (0.953–0.977)
Sodium	−0.086	0.038	5.031	0.025	0.918 (0.852–0.989)
Albumin	−0.062	0.030	5.031	0.039	0.939 (0.885–0.997)

### Machine learning results

**FIGURE 1:**
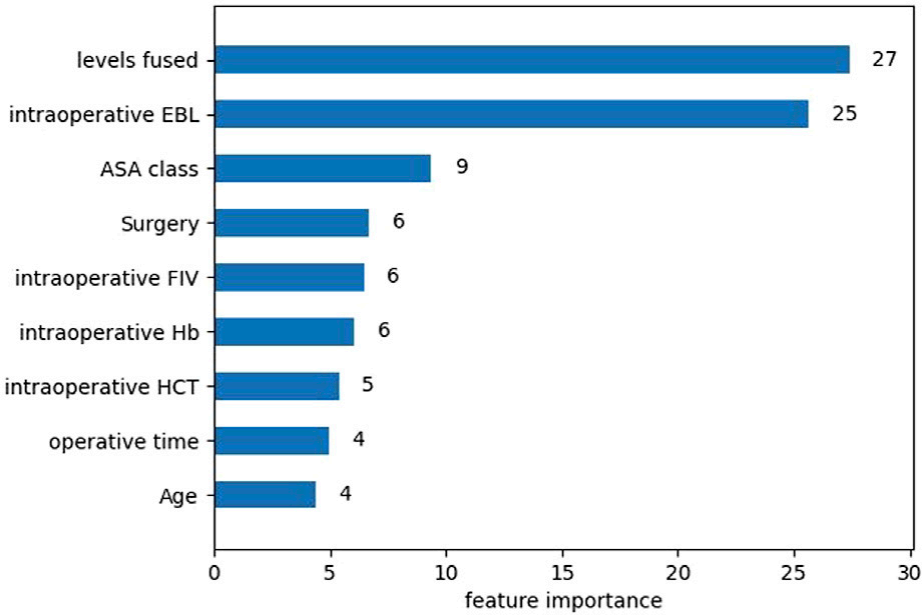
Importance score plot of the top nine features in the XGBoost model.

**FIGURE 2:**
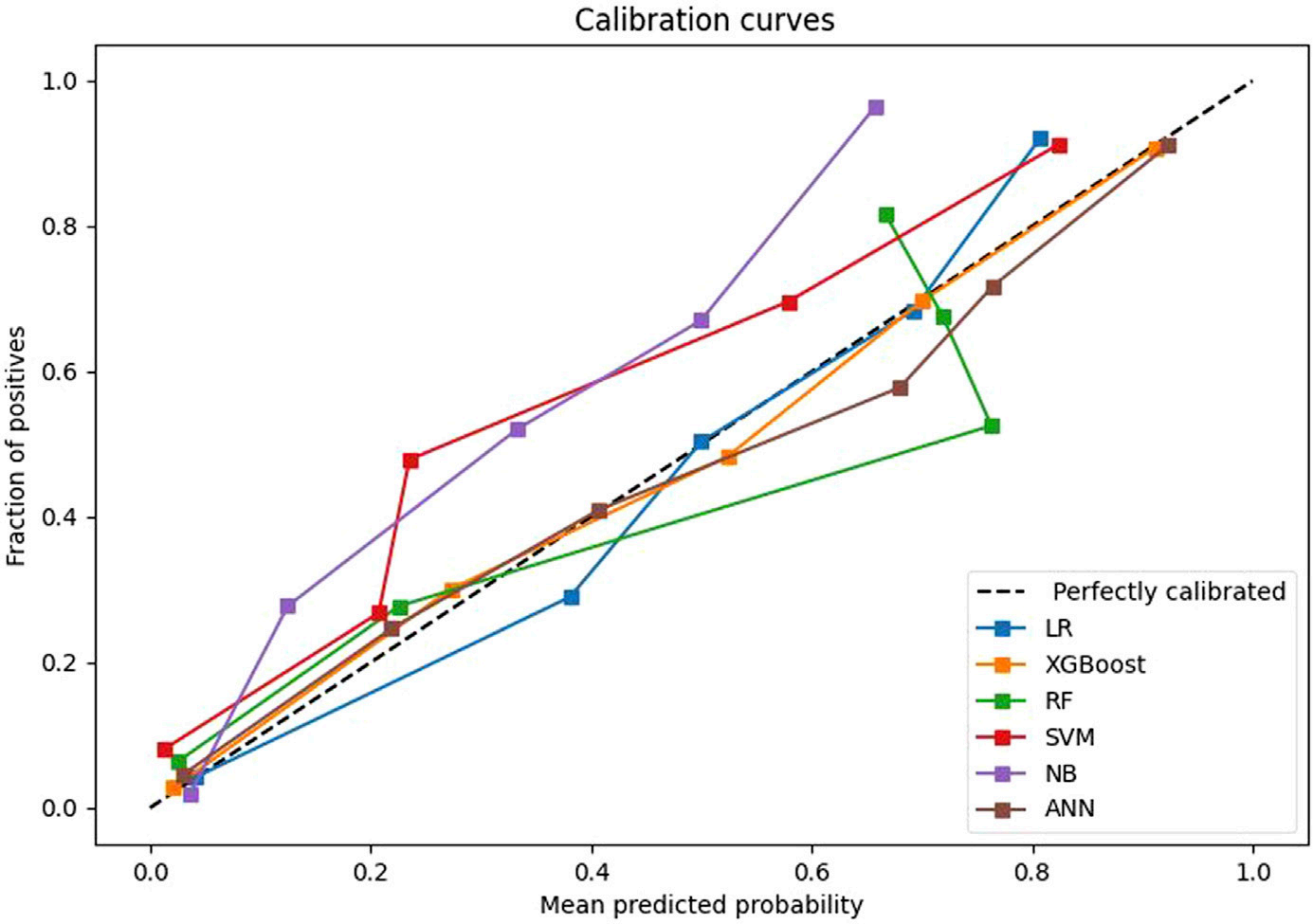
Comparison of calibration curves of the six models on validation set.

After univariate analysis, there were 23 significant features. In order to facilitate comparison with traditional logistic regression, the XGBoost algorithm was also applied to select the 9 features with top importance scores, including levels fused, total intraoperative blood loss, ASA class, surgery, intraoperative fluid infusion volume, preoperative Hb, preoperative hematocrit (HCT), operative time, and age (Figure 1). Five machine learning models were constructed based on the nine features. Among these models, the XGBoost model has the largest AUC value of 0.958, accuracy of 0.903, recall of 0.897, specificity of 0.904, F1-Score of 0.767, and precision of 0.670. all of them are higher than the Logistic regression model with AUC value of 0.930, accuracy of 0.866, recall of 0.882, specificity of 0.863, F1-Score 0.702, and precision 0.583 (Table 4). Furthermore, probabilistic calibration curves are utilized to assess the model’s performance. A superior model would possess a calibration curve situated close to the standard line. As can be observed in Figure 2, the Logistic Regression model shows a Sigmoid-like shape and is a lack of confidence. Whereas the ANN model illustrates an inverse Sigmoid shape and represents overconfidence. The RF, SVM and NB models gives even worse results, while the XGBoost model performs the optimal outcome with a calibration curve very close to the standard line.

**TABLE 4:** Comparison of the prediction performance of the six models on validation set.

Models	AUC	Accuracy	Recall	Specificity	F1-Score	Precision
LR	0.930	0.866	0.882	0.863	0.702	0.583
XGBoost	0.958	0.903	0.897	0.904	0.767	0.670
RF	0.932	0.874	0.882	0.872	0.714	0.600
SVM	0.933	0.861	0.882	0.856	0.694	0.571
NB	0.908	0.874	0.824	0.885	0.700	0.609
ANN	0.926	0.885	0.809	0.901	0.714	0.640

### Model explainability

**FIGURE 3:**
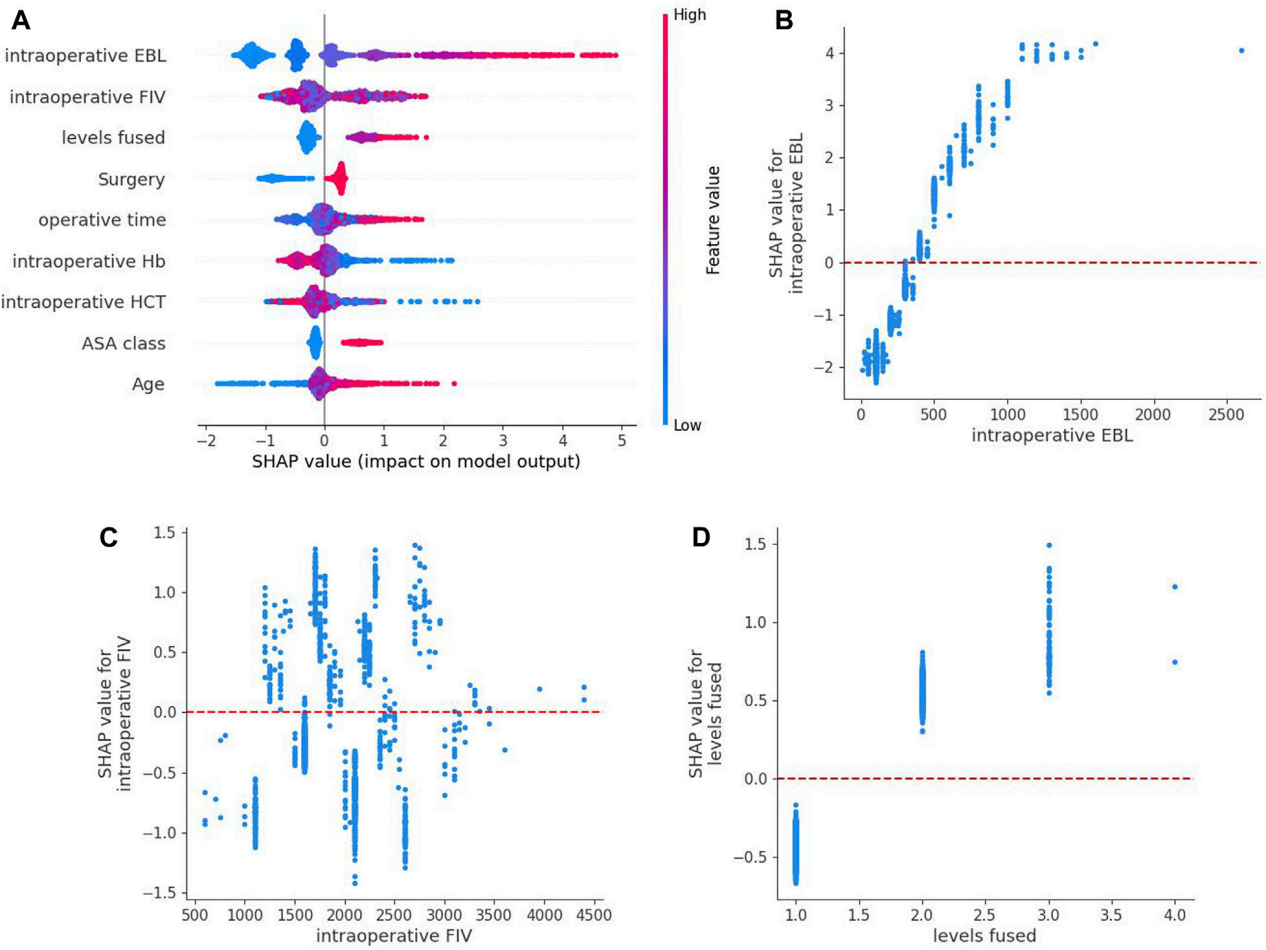
SHAP summary plot of the XGBoost model based on shapley additive explanations values. **(A)** An overview of the SHAP values for each feature in each sample. Each line represents a feature,a point represents a sample and the abscissa is the SHAP value. Red dots indicate higher feature values, whereas blue dots indicate lower feature values. **(B–D)** are SHAP dependence plots of the top 3 relatively important features output by the XGBoost prediction model. SHAP values for specific features exceed zero, representing an increased risk of blood transfusion.

Based on the above comparisons, we determined that the XGBoost model was the best prediction model for perioperative blood transfusion in posterior lumbar interbody fusion. We attempt to unlock the “black box” in the XGBoost model by SHAP values and explain how the model predicts blood transfusion. An overview of the SHAP values for each feature in each sample is shown in Figure 3A. The color represents feature values where the redder shade signifies a larger feature value. The figure shows that the risk factors for perioperative blood transfusion in posterior lumbar interbody fusion were total intraoperative blood loss, intraoperative fluid infusion volume, levels fused, operative time, ASA class, surgery, and age, while the protective factors were preoperative HCT and preoperative Hb. Additionally, SHAP dependence analysis was utilized to explore how individual features affected the output of the XGBoost prediction model. The SHAP dependence plots of the top 3 relatively important features output by the XGBoost prediction model are shown in Figures 3B–D. According to the figure, when the total intraoperative blood loss is higher than 400 mL and levels fused ≥2, the corresponding SHAP value is positive, thereby increasing the risk of blood transfusion for the patient. The intraoperative fluid infusion volume’s blood transfusion warning range is not particularly clear. Further detailed outcomes of SHAP dependence plots for the remaining six features are presented in the Supplementary Material.

**FIGURE 4:**
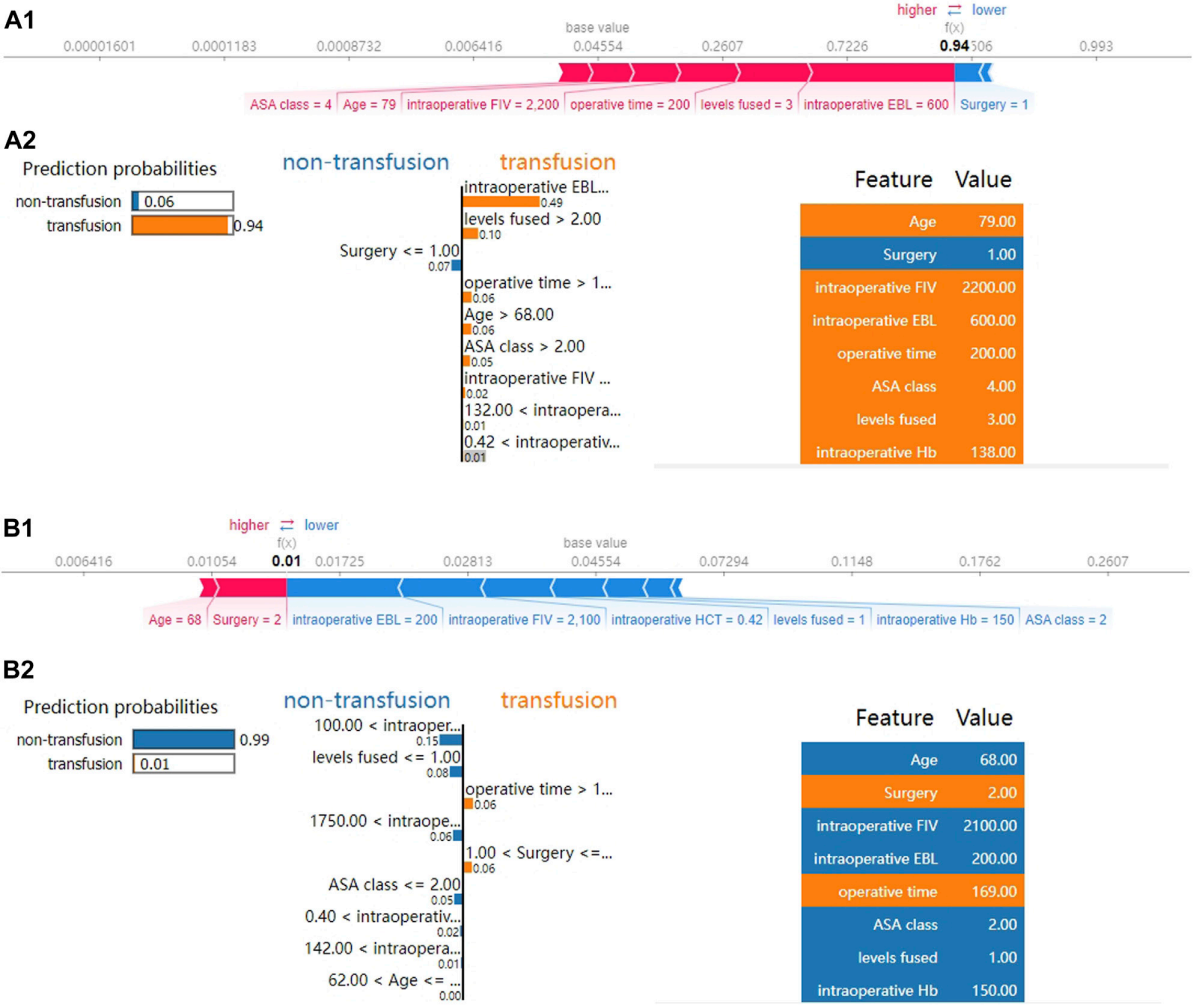
SHAP force analysis and local interpretable model-agnostic explanations (LIME) algorithm for explaining individual’s prediction results. **(A)** The true outcome is transfusion, and the predicted outcome is transfusion. **(B)** The true outcome is non-transfusion, and the predicted outcome is non-transfusion. **(A1, B1)**, the red and blue bars represent risk factors and protective factors, respectively; longer bars indicate greater importance of the feature. **(A2, B2)**, the left part of the figure shows the predicted outcomes using LIME. The middle part shows the critical values of the nine variables when they have the greatest effect on transfusion or non-transfusion. The length of each feature bar indicates the importance (weight) of that feature in the prediction. The right panel specifically lists the numerical sizes of the samples in these features.

Subsequently, we applied SHAP force analysis and the LIME algorithm to illustrate the individualized prediction of blood transfusion by extracting two samples from the validation set. Figures 4A1, A2 present a transfusion case of a 79-year-old man with a history of hypertension and coronary heart disease who was admitted to the hospital for lumbar spinal stenosis. The blood transfusion probability predicted by XGBoost model was 94%. The factors that increased the risk of blood transfusion included total intraoperative blood loss of 600 mL, levels fused of 3, operative time of 200 min, intraoperative fluid infusion volume of 2200 mL, age of 79 years, and ASA class Ⅳ. The factor that reduced the risk of blood transfusion was TLIF surgery. The XGBoost model predicted blood transfusion in this patient, and the actual result was also a transfusion. Similarly, Figures 4B1, B2 presents a non-transfusion case of a 44-year-old female admitted for lumbar spondylolisthesis with lumbar disc herniation. The XGBoost model predicted a 1% probability of blood transfusion. The patient’s total intraoperative blood loss of 200 mL, intraoperative fluid infusion volume of 2200 mL, levels fused 1, preoperative HCT of 0.42 L/L, and preoperative Hb of 150 g/L reduced the risk of blood transfusion, whereas PLIF surgery increased the risk of blood transfusion. The XGBoost model predicted no blood transfusion for this patient, which was the actual outcome.

## Discussion

Based on clinical data from patients undergoing posterior lumbar interbody fusion, classical logistic regression and five machine learning perioperative transfusion risk prediction models were built in this study. All prediction models ultimately included the 6 variables of surgery, ASA class, levels fused, total intraoperative blood loss, operative time, and preoperative Hb. This fully proves that the above six factors are important predictors of perioperative blood transfusion in lumbar posterior interbody fusion, which is basically consistent with the results of previous similar studies (Basques et al., 2015; Morcos et al., 2018; Jeremy et al., 2023). This study also compared the postoperative prognosis based on blood transfusion in patients undergoing lumbar posterior interbody fusion and showed that patients in the blood transfusion group had longer actual length of stay, higher total hospitalization costs, higher 90-day readmission rates, and higher incidence of complications within 90 days after surgery (Basques et al., 2014; Morcos et al., 2018).

According to this study, there is a higher chance of perioperative blood transfusion following PLIF surgery. Kunder et al. (de Kunder et al., 2017) and Lei F et al. (Lei et al., 2020) have demonstrated that intraoperative bleeding in TLIF surgery is significantly lower than in PLIF surgery, potentially due to differences in anatomical regions. The classical PLIF technique involves removal of the ligamentum flavum and complete removal of the posterior lamina to access the intervertebral space via extensive laminotomy (Cloward, 1953). However, TLIF technique is a modification of the PLIF technique, which involves removal of only one side of the facet joints to access the posterolateral intervertebral discs via a unilateral foraminal approach (Lowe and Tahernia, 2002), preserving the other side of the facet joints, vertebral plates, and posterior ligaments of the spine. Therefore, the TLIF technique may be associated with fewer complications, shorter operative time, and less blood loss than the PLIF technique. Patients with ASA class 3 and above also have increased transfusion rates, wherein higher ASA classes indicate more medical comorbidities. Patients with considerable comorbidities tend to have lower reserves and lower transfusion thresholds (Morcos et al., 2018). Levels fused, total intraoperative blood loss and operative time are also risk factors for perioperative blood transfusion (Aoude et al., 2016a; Durand et al., 2018; Wang et al., 2021a). The lumbar spine is richly endowed with blood vessels, mainly including the internal vertebral venous plexus and the external vertebral venous plexus, to collect venous blood from the spinal cord, spine and soft tissues. The more the number of fusion segments in spinal fusion, the more extensive stripping of paravertebral muscles and soft tissues is required for pedicle nail placement and intravertebral decompression. Owing to the abundant blood vessel distribution in the lumbar vertebrae, a larger operation scope necessitates more operations, thereby prolonging the operation time and correspondingly increasing blood loss in the body. Hence, carefully inquiring the medical history, performing a physical examination and analysis of imaging data, grasping the indications of fusion surgery, and elucidating the responsible segments for precise lumbar fusion surgery are effective ways to lower the risk of perioperative blood transfusion. Notably, a lower preoperative hemoglobin level also raises the perioperative transfusion rate. The lower the preoperative red blood cell count, hemoglobin and hematocrit, the worse the ability to compensate for intraoperative bleeding, and the greater the likelihood of perioperative blood transfusion. This suggests that orthopedic surgeons should focus on improving hemoglobin levels before surgery, which can reduce the risk of intraoperative and postoperative blood transfusion (Liu et al., 2021).

Furthermore, five machine learning predictive models were developed based on selected features of the XGBoost algorithm. The performance of each model was evaluated on the validation set data. The results showed that the XGBoost model had the best prediction effect, with an AUC value of 0.958, small differences in the accuracy, recall, specificity, F1-Score, and precision, and the calibration curve was closest to the standard line. The superior predictive performance of the XGBoost model is primarily attributed to: The XGBoost algorithm is a nonlinear integrated learning algorithm. Its tree model has the ability to infinitely split, thus allowing for infinite approximation of the Vapnik-Chervonenkis dimension and improving the accuracy of data fitting. To address the issue of overfitting caused by high dimensions, the algorithm utilizes the L1 and L2 regularization method. Additionally, the modelling retains correlation among variable features, enhancing the model’s predictive effectiveness. By contrast, the logistic regression model in this study had an AUC value of 0.930, and its accuracy, recall, balanced F-score, precision, and calibration curve performance was inferior to that of the XGB model. One possible explanation for this could be the model’s low computational complexity, which is liable to cause underfitting and a low classification accuracy. However, it is worth noting that the predictive performance of the logistic regression model in this study was significantly better than that of the nomogram model (AUC value 0.890) reported in previous literature (Liu et al., 2021). The RF and SVM models exhibit moderate predictive capabilities. Random Forest comprises numerous decision trees resulting in high computational complexity and dependence on vast training datasets for improved prognostication. While support vector machine lacks a universal approach to nonlinear difficulties, occasionally necessitating the identification of an appropriate kernel function. The prediction performance of the ANN model is slightly inferior, possibly because the neural network requires a large number of parameters and protracted learning time. This can lead to local minima or even a failure to achieve the learning objective. Whereas the NB model has the worst prediction performance, probably due to the use of the assumption of sample attribute independence, so its effect is not good if the sample attributes are correlated. Therefore, the prediction model based on XGBoost algorithm has great potential in the prediction of perioperative blood transfusion in posterior lumbar interbody fusion. Several previous studies across various patient populations have also demonstrated the efficacy of XGBoost in disease prediction studies, highlighting the model’s widespread applicability (Hu et al., 2022; Ma et al., 2022; Fan et al., 2023).

Moreover, machine learning’s usefulness is limited by the fact that they often exhibit “black box” performance that is challenging to interpret (Watson et al., 2019; Azodi et al., 2020). To address this issue, this study uses the SHAP algorithm, a post-hoc interpretable technique for machine learning models, to perform global interpretive analysis and personalised attribution analysis of nine risk features in the XGBoost model, and further proposes the warning range of the risk features. At the same time, two specific instances are selected for visual prediction based on LIME algorithm, which is easier to be understood and used by clinical practitioners.

This study also has several limitations. Firstly, it is a retrospective study conducted in a single large-capacity center with missing or incomplete data, which may introduce selective bias and weaken statistical test efficacy. In future studies, prospective randomized controlled studies will be conducted to further confirm the present findings and for external validation, especially in other regions and countries. Secondly, the XGBoost classifier has many parameters, and fine-tuning it with the grid search method is inefficient. The performances of the classifier in this work may depend largely on the results of feature selection, and the effects of different feature selection methods on model performance will be further investigated in future studies.

Conclusively, this study has retrospectively analyzed clinical data to construct the XGBoost prediction model. This model can predict perioperative blood transfusion in lumbar posterior interbody fusion, which can assist orthopedic surgeons in enhancing their surgical transfusion decision-making efficiency. It is worthy of promotion and application in the clinic. No research has been found to have utilised machine learning models to predict the risk associated with perioperative blood transfusion in posterior lumbar interbody fusion, beyond traditional logistic regression modelling. This is the first study to construct and compare multiple machine learning models for individualized prediction of perioperative blood transfusion based on the clinical data of patients undergoing posterior lumbar interbody fusion.

## Data Availability

The original contributions presented in the study are included in the article/Supplementary Material, further inquiries can be directed to the corresponding author.
