# Supplementary material for: Predictive modeling of perioperative blood transfusion in lumbar posterior interbody fusion using machine learning
Source: Front Physiol. 2023 Dec 22;14:1306453. doi: 10.3389/fphys.2023.1306453 (PMC10767743; doi:10.3389/fphys.2023.1306453)
Supplement: Supplementary file 1 [file DataSheet1.docx]

Supplementary Material

Table S1. Hyperparameter configuration for algorithms.

| Algorithm name | parameter values |
| --- | --- |
| LR | Penalty = L1, C = 0.3 |
| XGBoost | learning_rate = 0.05, n_estimators = 300, max_depth = 3, min_child_weight = 1, gamma = 0.5, colsample_bytree = 0.7, subsample = 0.9, reg_lambda = 0.05, reg_alpha = 0.1 |
| RF | n_estimators=500, max_depth=4, n_jobs=-1, criterion='gini' |
| SVM | kernel = "linear", C = 10 |
| NB | default parameters |
| ANN | neurons=7, dropout = 0.3, epochs=600, batch_size=10 ,  loss = 'binary_crossentropy', optimizer = 'adam', metrics = 'accuracy' |

LR: logistic regression, XGBoost: eXtreme Gradient Boosting, RF: random forest, SVM: support vector machine, ANN: artificial neural network

# Supplementary Figures


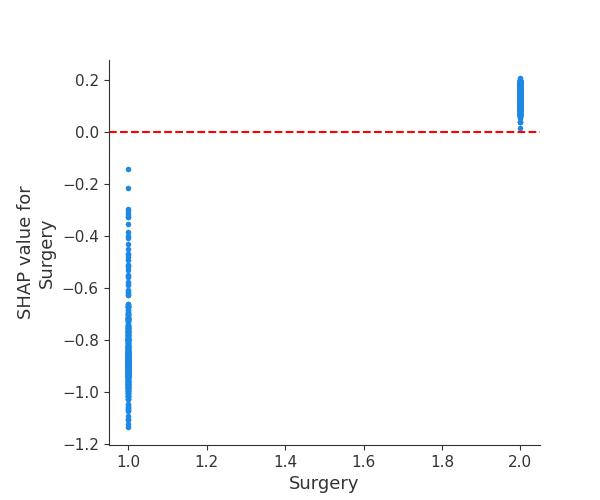
Figure S1: SHAP dependence plots for surgery


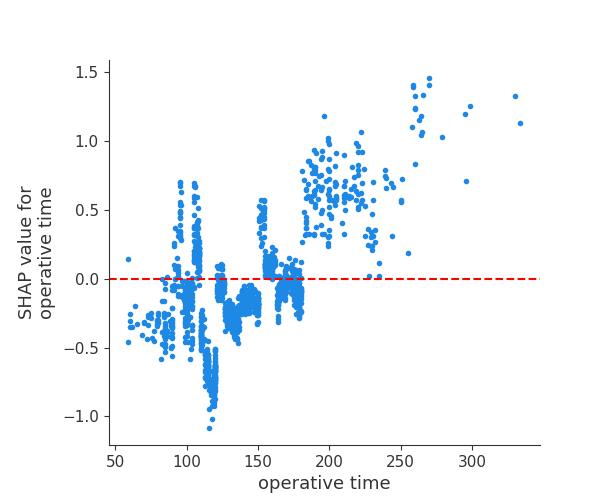


Figure S2: SHAP dependence plots for operative time


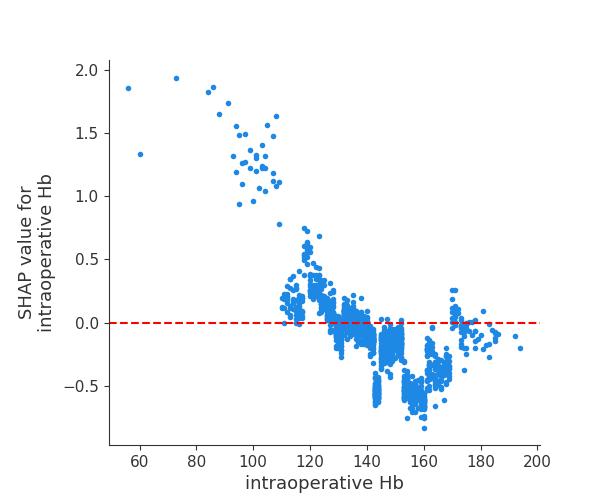

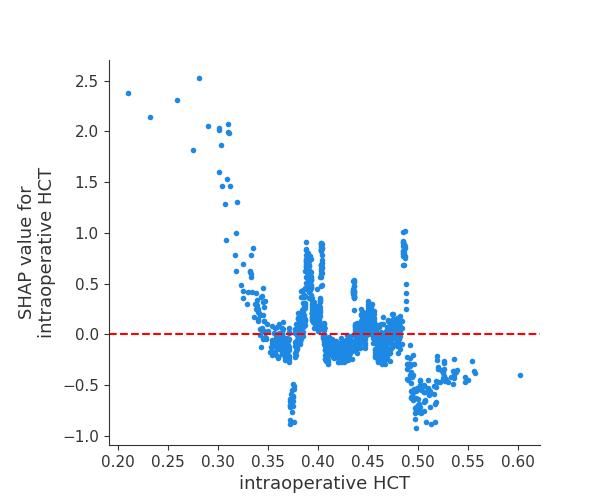
Figure S3: SHAP dependence plots for intraoperative Hb


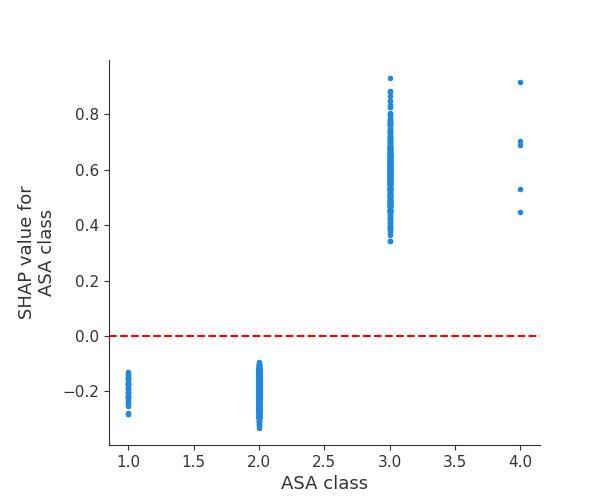
Figure S4: SHAP dependence plots for intraoperative HCT

Figure S5: SHAP dependence plots for ASA class


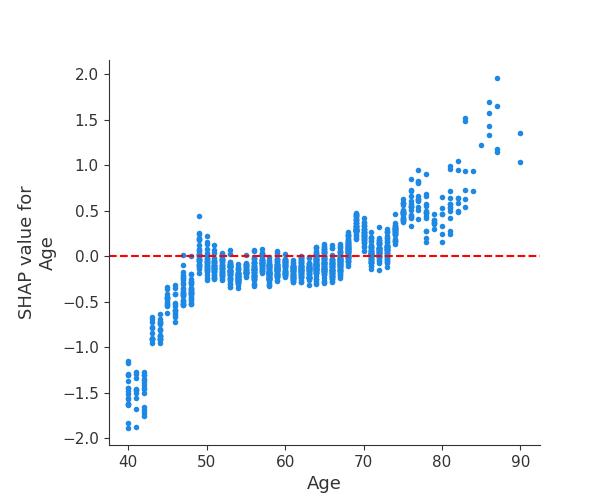
Figure S6: SHAP dependence plots for Age
